# Pelvic Primary Staphylococcal Infection Presenting as a Thigh Abscess

**DOI:** 10.1155/2013/539737

**Published:** 2013-04-02

**Authors:** T. O. Abbas

**Affiliations:** General Surgery Department, Hamad General Hospital, Doha 3050, Qatar

## Abstract

Intra-abdominal disease can present as an extra-abdominal abscess and can follow several routes, including the greater sciatic foramen, obturator foramen, femoral canal, pelvic outlet, and inguinal canal. Nerves and vessels can also serve as a route out of the abdomen. The psoas muscle extends from the twelfth thoracic and fifth lower lumbar vertebrae to the lesser trochanter of the femur, which means that disease in this muscle group can migrate along the muscle, out of the abdomen, and present as a thigh abscess. We present a case of a primary pelvic staphylococcal infection presenting as a thigh abscess. The patient was a 60-year-old man who presented with left posterior thigh pain and fever. Physical examination revealed a diffusely swollen left thigh with overlying erythematous, shiny, and tense skin. X-rays revealed no significant soft tissue lesions, ultrasound was suggestive of an inflammatory process, and MRI showed inflammatory changes along the left hemipelvis and thigh involving the iliacus muscle group, left gluteal region, and obturator internus muscle. The abscess was drained passively via two incisions in the posterior left thigh, releasing large amounts of purulent discharge. Subsequent bacterial culture revealed profuse growth of *Staphylococcus aureus*. The patient recovered uneventfully except for a moderate fever on the third postoperative day.

## 1. Introduction

Intra-abdominal infections may reach extra-abdominal sites by traveling via certain well-defined routes [1], presenting as abscesses in extra-abdominal locations [2], including the buttock [3, 4], thigh [5], and calf [6]. Four main abdominal sources have been reported: intestinal, renal, vertebral, and iliopsoas muscle [6]; diabetes mellitus, trauma, and immunodeficiency are predisposing factors [6]. 

The greater sciatic foramen, obturator foramen, femoral canal, pelvic outlet, and inguinal canal all have the potential to allow communication between the abdomen and the thigh or perineum [5, 7]. Because the psoas muscle extends from the twelfth thoracic and fifth lower lumbar vertebrae to the lesser trochanter of the femur, disease in this muscle can track dependently directly along the muscle, out of the abdomen, and appear as a thigh abscess [3, 4, 8]. The condition is rare but carries a high mortality rate if not diagnosed early [6, 9]. However, the symptoms are often vague and can be ascribed to the thigh abscess itself, often leading to a lack of further diagnostics to rule out an intra-abdominal source. Generally, patients with a thigh abscess secondary to an intra-abdominal source present with general malaise, usually a fever, leukocytosis, and sometimes anemia, especially if the progression is chronic [4–6, 9, 10]. Increased C-reactive protein has also been reported [10]. Computed tomography (CT) scans are the most useful diagnostic tool, but radiographs, ultrasound, and magnetic resonance (MR) imaging also provide useful information [8]. An air-fluid interface can be seen on CT scans of the abdomen, suggesting a gas-producing abscess [6]. Thigh abscesses are rare but well documented as primary presentations in patients with intra-abdominal sepsis. 

A patient is described with a primary staphylococcal pelvic infection presenting as a left thigh abscess. This case is novel in that the causative organism was atypical and the primary source of the staphylococcal infection was unknown. Furthermore, the abscess was tracked through the obturator foramen, presenting in the posterior thigh, distant from the original perirectal infection.

## 2. Case Report

A 60-year-old man presented to the emergency room with a 5-day history of severe left posterior thigh pain associated with loose motions. The patient also had fever and chills. His medical history was unremarkable, with no history of diabetes mellitus or abdominal surgery. On physical examination, the patient was sweating and appeared ill. His temperature was 36.6°C, his blood pressure was 14.532 kPa, and his pulse rate was 105 beats per minute. The posterior aspect of his left thigh was diffusely swollen and exquisitely tender to the touch, with no palpable crepitance. The skin overlying the posterior thigh, extending from the knee to the lower gluteal fold, was erythematous, shiny, and tense but not indurated. A distal neurovascular examination was normal. His Hb was 13.8 g/L, total leukocyte count was 18800 × 10^9^/L, and a renal function profile was normal.

**Figure 1:**
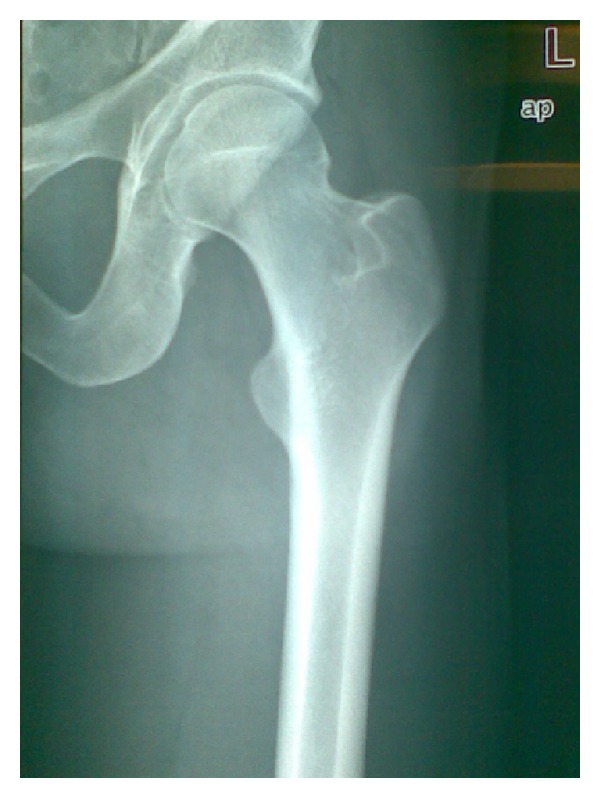
Plain X-ray of the thigh showing no significant soft tissue abnormalities.

**Figure 2:**
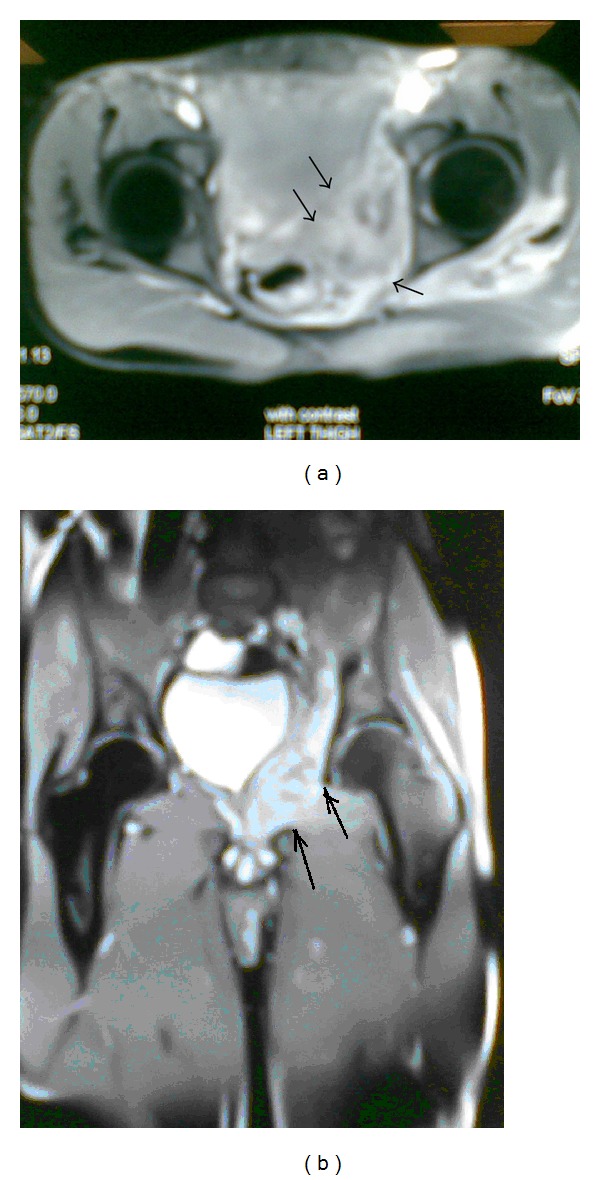
An MRI of the patient showing inflammatory changes along the left hemipelvis (indicated with arrows in (a)) and thigh extending from the iliacus muscle through the iliacus muscle group, along with conglomerations of small loculated fluid collections under the skin of the left gluteal region and along the medial aspect of the left hemipelvis abutting the obturator internus muscle. (b) Arrows indicate the borders of the obturator foramen.

An X-ray of his thigh showed no significant soft tissue lesions (Figure 1). Ultrasound revealed signs of inflammation but no signs of fluid in the abdomen. MR imaging showed inflammatory changes along the left hemipelvis and thigh. These changes extended from the iliacus muscle through the iliacus muscle group, together with a conglomeration of small loculated fluid collections under the skin surface of the left gluteal region and along the medial aspect of the left hemipelvis abutting the obturator internus muscle (Figure 2).

Two separate incisions were made on the posterior aspect of the left thigh and another incision medially, resulting in the drainage of large amounts of pus, which appeared to track along the musculofascial planes from above deep into the thigh. The thigh abscess was continuous with the original perirectal/retroperitoneal abscess and tracked through the obturator foramen. Passive drainage was effective. Bacteriological examination of the pus showed profuse growth of *Staphylococcus aureus*. The patient received ceftriaxone and metronidazole antibiotic therapy intravenously for 10 days in addition to percutaneous drainage. An original cause for the abdominal abscess was not determined.

The patient's postoperative period was uneventful, but he experienced a moderate degree of fever on the third postoperative day. The blood pressure, Hb, total leukocyte count, and renal function profiles were reassessed in the postoperative period and found to be within normal limits. The wound was closed secondarily. 

## 3. Discussion 

Traditionally, abscesses of the thigh appear to arise primarily from local structures. Among the most common causes are skin and soft tissue infections, osteomyelitis, infected posttraumatic hematoma, thrombophlebitis, and pyomyositis [11]. Abscesses arising from pelvic contents may present with signs and symptoms in remote locations, distant from the abdomen [12]. Pelvic infections can be primary, as in psoas abscesses. Although the source of infection may be unknown, these infections are thought to arise by hematogenous spread or secondary to an adjacent retroperitoneal or intra-abdominal infection. Other causes of a secondary abscess include appendicitis, diverticulitis, ulcerative colitis, osteomyelitis, neoplasm, disk infection, renal infections, and trauma [13]. Certain intra-abdominal inflammatory pathologies may be involved in the etiology of painful, swollen thighs, including diverticulitis, acute appendicitis, colorectal carcinoma, Crohn's disease, ischiorectal abscess, rectal trauma, and primary staphylococcal abscess [2, 14–16]. 

A review of reported cases suggests that intra-abdominal sepsis may spread into the thigh through direct soft tissue extension or through natural abdominal wall defects, mainly along the femoral canal, obturator foramen, sacrosciatic notch, or the psoas muscle behind the inguinal ligament and iliofemoral vessels [13, 17]. Up to 14% of retroperitoneal abscesses are considered primary because no other associated condition can be found. Recently, retroperitoneal abscesses have been described as late complications originating from “lost” stones following laparoscopic cholecystectomy [6]. The most common pathogen in a primary psoas abscess is *S. aureus* (88.4% of cases), with other pathogens including *Streptococci *species (4.9%), *Escherichia coli *(2.8%) [18], *Pasteurella multocida*, *Proteus *species, *Mycobacterium tuberculosis*, *Bacteroides *species, *Clostridium welchii*, *Yersinia enterocolitica*, and *Klebsiella *species [19, 20].

Because of its often insidious onset and subtle clinical signs in retroperitoneal abscess, the correct diagnosis may be delayed in many patients [17]. Generally, an abscess will be located on the same side as its source, limiting the differential diagnosis and allowing for a more focused investigation [1]. Radiological abnormalities are reported in 40–90% of patients with retroperitoneal abscesses [14, 17]. The presence of fluid collections on abdominal ultrasound is also of diagnostic importance. Chest X-rays may reveal elevation or fixation of the diaphragm, pleural effusion, and/or basal atelectasis. Similarly, the presence of a retroperitoneal abscess may be indicated by abnormal psoas shadow, scoliosis, or a soft tissue mass on plain abdominal roentgenograms [17].

Drainage can be performed surgically or radiologically. Percutaneous drainage may be difficult in some patients because of the location of the abscess but should be employed whenever possible. Even in patients with complex, multiloculated abscesses, percutaneous drainage should be attempted, with open surgical drainage reserved only for patients in whom percutaneous drainage fails. Patients with secondary psoas abscesses require correction of their underlying disease in addition to the drainage procedure. Extraperitoneal drainage is a safe, effective method of draining these abscesses [9].

Drainage can be either direct or percutaneous. Although abscesses inside the thigh are due to direct extension from the retroperitoneum, it may be better to make a separate incision on the thigh to drain the abscess rather than draining from the trunk. Draining a thigh abscess from an incision at the thigh has two advantages. First, the abscess can be more easily and directly approached. Second, the viability of the muscle and fascia of the thigh, as well as the need for further debridement, can be adequately evaluated [21]. Indeed, some thigh abscesses can be cured by drainage alone [5, 22, 23]. 

Initially, percutaneous abscess drainage was limited to simple abscesses (i.e., well-defined, unilocular) with safe drainage routes, but drainage was later expanded to include complex abscesses (i.e., loculated, ill-defined, or extensively dissecting abscesses), multiple abscesses, abscesses with enteric fistulas or whose drainage routes traversed normal organs, and complicated abscesses (i.e., appendiceal, splenic, interloop, and pelvic) [23]. 

Retroperitoneal abscesses can be treated with intravenous antibiotics alone but only if the abscess is small (<3 cm) and the patient's general condition is good. Drainage, however, is required in most cases. The initial procedure of choice is ultrasound- or CT-guided percutaneous drainage, which has a high success rate (>80%), although the insertion of more than one catheter is sometimes necessary. Surgical exploration should be reserved for abscesses that do not drain adequately during percutaneous drainage or when malignancy in either the urinary tract or the bowel is suspected. Collections tracking along the psoas fascia into the lower limb, as in our patient, should be drained by several separate incisions in conjunction with debridement [1].
